# Robust PVC Identification by Fusing Expert System and Deep Learning

**DOI:** 10.3390/bios12040185

**Published:** 2022-03-22

**Authors:** Zhipeng Cai, Tiantian Wang, Yumin Shen, Yantao Xing, Ruqiang Yan, Jianqing Li, Chengyu Liu

**Affiliations:** 1 School of Instrument Science and Engineering, Southeast University, Nanjing 210096, China; zhipeng@seu.edu.cn (Z.C.); 220183259@seu.edu.cn (T.W.); 220213661@seu.edu.cn (Y.S.); 230198304@seu.edu.cn (Y.X.); 2 School of Mechanical Engineering, Xi’an Jiaotong University, Xi’an 714009, China; liq@seu.edu.cn

**Keywords:** electrocardiogram, K-means clustering algorithm, premature ventricular contraction, rule-based decision algorithm

## Abstract

Premature ventricular contraction (PVC) is one of the common ventricular arrhythmias, which may cause stroke or sudden cardiac death. Automatic long-term electrocardiogram (ECG) analysis algorithms could provide diagnosis suggestion and even early warning for physicians. However, they are mutually exclusive in terms of robustness, generalization and low complexity. In this study, a novel PVC recognition algorithm that combines deep learning-based heartbeat template clusterer and expert system-based heartbeat classifier is proposed. A long short-term memory-based auto-encoder (LSTM-AE) network was used to extract features from ECG heartbeats for K-means clustering. Thus, the templates were constructed and determined based on clustering results. Finally, the PVC heartbeats were recognized based on a combination of multiple rules, including template matching and rhythm characteristics. Three quantitative parameters, sensitivity (Se), positive predictive value (P+) and accuracy (ACC), were used to evaluate the performances of the proposed method on the MIT-BIH Arrhythmia database and the St. Petersburg Institute of Cardiological Technics database. Se on the two test databases was 87.51% and 87.92%, respectively; P+ was 92.47% and 93.18%, respectively; and ACC was 98.63% and 97.89%, respectively. The PVC scores on the third China Physiological Signal Challenge 2020 training set and hidden test set were 36,256 and 46,706, respectively, which could rank first in the open-source codes. The results showed that the combination strategy of expert system and deep learning can provide new insights for robust and generalized PVC identification from long-term single-lead ECG recordings.

## 1. Introduction

Cardiovascular diseases (CVDs) are the foremost cause of human death worldwide, which can lead to over 31% of deaths every year. With the progressive aging of populations worldwide, the number of patients with CVDs may continue to increase. It is estimated that the number of deaths due to CVDs will increase from 17 million in 2016 to 24 million in 2030 [1]. Therefore, monitoring and preventing CVDs in advance has become one of the important tasks for many countries [2].

Arrhythmia is a common CVDs, which refers to a series of rhythm and/or waveform irregular. As one of the most common arrhythmias, premature ventricular contraction (PVC) is caused by premature ectopic beats in the right or left ventricle [3]. Frequent PVC and multisource PVC detection have important clinical significance [4]. Clinicians generally detect PVC by observing rhythmic changes and subtle morphological changes from electrocardiogram (ECG) signal. However, this visual inspection may increase the manual interpretation work for physicians and lead to low efficiency for long-term PVC recognition. In order to reduce the workload of clinicians and improve PVC detection accuracy, researchers developed computer-aided systems for automagical diagnosis [5].

Various automatic ECG heartbeat classification algorithms have been developed in recent decades, which can be summarized into two categories: expert system (ES)-based and deep learning (DL)-based methods. The ES-based methods classify heartbeats into different categories by judging multiple features with fixed thresholds. Most ES-based algorithms utilize rule-based features derived from rhythmic intervals (RR-interval, QT-interval, PR-interval, etc.) and morphological characteristics (P-wave, Q-wave, T-wave, etc.). Liu et al. [6] presented a personalized ECG template construction method and detected PVC beats based on template matching, and the sensitivity (Se) on the MIT-BIH arrhythmia database (MIT-BIH-AR) (DS2) reached over 99%. Although this method has low computational complexity and can be applied for real-time conditions, the high performance is not tested on other databases especially on the dynamic noisy signals. Nahar et al. [7] proposed an algorithm for PVC detection based on morphological transformation and cross-correlation technology, which used the morphological features to directly detect PVC. The potential of this proposed method was examined using 32 records from the MIT-BIH-AR database, reporting a specificity (Sp) of 96.67%, and a Se of 95.2%. Li et al. [8] proposed a low-complexity data-adaptive approach for PVC recognition. They tested the method on INCART database and achieved a Se of 93.4%, an accuracy (ACC) of 94%, and a positive predictive value (P+) of 66.5%. These methods can be used for real-time applications without patient-specific consideration, as these methods have low computational complexity and good generalization capabilities. However, they need professional researchers to choose features and specific thresholds according to different tasks. Moreover, these detailed features are susceptible to noise interference, resulting in poor anti-noise ability of the algorithm.

With the development of machine learning, numerous DL-based methods have been developed, including auto-encoding (AE) [9], convolutional neural network (CNN) [10], block-based neural network (BBNN) [11], long-short term memory (LSTM) [12], support vector machine (SVM) [13], decision tree [14], cascade forward neural network (CFNN) [15], and random forest [16], etc. The DL-based method omits the handcrafted features extraction process, as the DL network can automatically extract the high-dimensional features. Therefore, DL-based methods can be applied in situations with big data processing capabilities, such as cloud computing platforms [17]. Yildirim et al. [1] presented a new 1D-convolutional neural network model for cardiac arrhythmia detection based on long-duration ECG signal analysis, which achieved an ACC of 91.33% for 17 cardiac arrhythmia classes classification in the MIT-BIH-AR database. Similarly, Pławiak et al. [18] proposed genetic ensembles of SVM-based classifiers for the same classification task and achieved a Se of 91.40% and an ACC of 98.99%. These two methods can be used for real-time signal processing and cloud computing on mobile devices, as they eliminate the need for detection and segmentation of QRS complexes. However, neither of these two methods can classify ECG segments that contain multiple ECG abnormalities. Shadmand et al. [11] employed the particle swarm optimization algorithm to optimize the structure and weights of BBNN and obtained an accuracy of 97.00% for five classes of ECG classification on the MIT-BIH-AR database. This method highly relied on large volumes of labeled data and computing resources to obtain its satisfactory performance on different databases.

Although the reported ES- and DL-based automatic heartbeat classification algorithms can achieve high performances on different databases, the extracted features of ES-based method require professional knowledge and are susceptible to noise; while the DL-based method is unexplainable and is easy to overfit on a small amount of labeled data. Therefore, in order to ensure the accuracy of ES-based and DL-based algorithms while considering the disadvantages of these two methods, a robust PVC identification algorithm based on a novel expert system and deep learning combination strategy was proposed in this paper. To evaluate its performance and generalization capacity, the method was tested on three different databases: the MIT-BIH-AR database, the St. Petersburg Institute of Cardiological Technics (INCART) database and the China Physiological Signal Challenge 2020 (CPSC2020) database. There are three major contributions of the proposed work. (1) This article proposed a novel expert system and deep learning combination strategy for PVC recognition in single-lead ECG. (2) The developed PVC detection algorithm is unsupervised, since the employed LSTM-AE network is used as the feature extraction process for heartbeat clustering. (3) The designed method is less complex and lightweight compared to most of the proposed automatic PVC detection methods.

## 2. Materials and Methods

### 2.1. MIT-BIH-AR Database

The lead II ECG signal of MIT-BIH arrhythmia (MIT-BIH-AR) database is used as the training set in our study. The database contains 48 half-hour two channel ambulatory ECG recordings, obtained from 47 subjects, and sampled at 360 Hz. Following the Association for the Advancement of Medical Instrumentation (AAMI) recommendations, the experiments are performed by excluding four records (102, 104, 107, and 217) containing paced beats, and the remaining 44 recordings are used as training set. Similar to [19], the fusion and supraventricular beats are treated as Non_PVC beats while unclassified (Q) and distortion beats are ignored, so there are 6990 PVC beats and 92,851 Non_PVC beats (Table 1).

### 2.2. INCART Database

The performance of the proposed algorithm was evaluated on the INCART database, which consists of 75 12-lead ECG records. Each recording was sampled at 275 Hz and 30 min in duration. The annotations were produced by an automatic algorithm and then corrected manually, containing over 175,000 annotations in total [15]. Among these recordings, ECGs of lead II are adopted as our experimental data [20], and the ventricular ectopic beats (V) are regarded as PVC beats, and the others are Non_PVC beats.

### 2.3. CPSC2020 Database

CPSC2020 database is a wearable ECG database constructed for challenging PVC and supraventricular premature beat detection tasks [21], including pathological arrhythmias and poor signal quality due to artifact and noise. The training data consists of 10 single-lead ECG recordings collected from arrhythmia patients, each of the recording lasts for almost 24 h. The test set contains similar ECG recordings, which are not public. All data were collected with a sampling frequency of 400 Hz. It is worth noting that we did not participate in CPSC2020 in order to avoid doubts (we are affiliated with the organizer), but we tested our algorithm on this database and compared it with the top five teams.

## 3. Method

**Figure 1:**
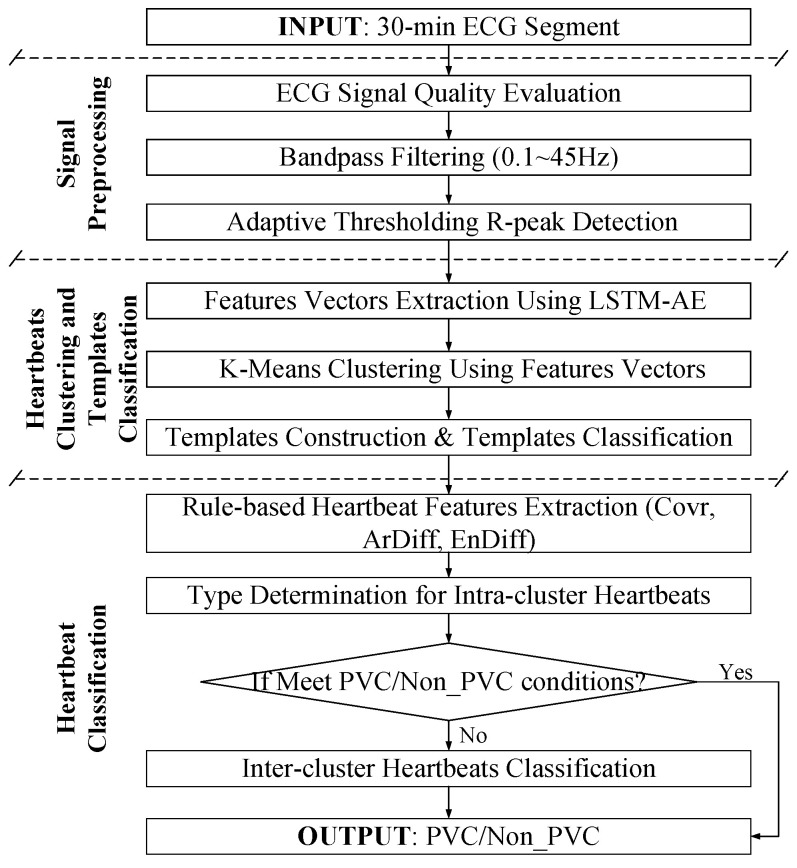
Flowchart of proposed method.

In this study, ECG recordings were cut into 30 min ECG segments. Each 30 min ECG segment was preprocessed to exclude the noise episodes and filter the artifacts for accurate R-peak detection. Thereafter, the feature vectors extracted by LSTM-AE were used for template construction based on K-means clustering, and the type of each template was determined by rule-based method. Finally, PVC heartbeats were identified by several rules. The flowchart of the proposed method is illustrated in Figure 1.

### 3.1. Signal Preprocessing

ECG signal is easily polluted by a variety of noises, including body movement, ECG-lead off, etc. The corrupted ECG data could significantly affect the PVC identification. To remove the unacceptable ECG segments with poor signal quality, the signal quality assessment is used based on our previous work [22]. In brief, seven signal quality indices (SQIs) were calculated to train an SVM-based signal quality classification model, the training strategy and parameters setting were same as our previous work. After that, the baseline drift and high-frequency noise is excluded by a Butterworth band-pass (0.1–45 Hz) filter. Then, R-peaks are detected using an adaptive and time-efficient algorithm [23]. It was an adaptive method integrating wavelet-based multiresolution analysis, signal mirroring, local maximum detection, and amplitude and time interval thresholding. The R peaks were refined three times by replacing the detected R peak with the position of its surrounding (±25 ms) maximum absolute amplitude to address the R-peak misalignment problem. Finally, the 30 min ECG segment is divided into ECG heartbeats with 0.5 s length window centered around the detected R-peaks (0.1 s in front and 0.4 s after) referred from previous works [24].

### 3.2. Heartbeats Clustering and Templates Classification

#### 3.2.1. Feature Vectors Extraction Based on LSTM-AE

**Figure 2:**
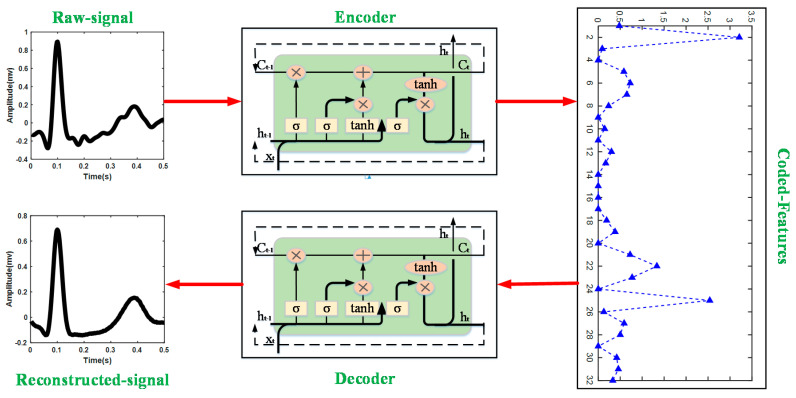
Structure of LSTM-AE in this study.

The long short-term memory-based autoencoder (LSTM-AE) network is used to extract the feature vectors of ECG heartbeats in this research. Figure 2 shows the structure of LSTM-AE. LSTM is designed for processing time series based on the framework of the recurrent neural network, consisting of three gate structures: input gate, forget gate, and output gate. The forget gate decides what information will be thrown away from the previous cell state. The vectors $f_{t}$ generated by the hidden state $h_{t-1}$ from the previous LSTM cell and the input $x_{t}$ of the current step *t*. The generation process can be represented as

(1) $$f_{t}=\sigma \left(W_{f}\cdot \left[h_{t-1},x_{t}\right]+b_{f}\right)$$

where $W_{f}$ is the weighted matrix of the forget gate and $b_{f}$ is the bias. As for the input gate, the vector $i_{t}$ and the input candidate information $\tilde{C_{t}}$ is also generated by the hidden state $h_{t-1}$ and the input $x_{t}$ as

(2) $$i_{t}=\sigma \left(W_{f}\cdot \left[h_{t-1},x_{t}\right]+b_{i}\right)$$

(3) $$\;\tilde{C_{t}}=\sigma \left(W_{C}\cdot \left[h_{t-1},x_{t}\right]+b_{C}\right)$$

The weighted matrices of $W_{i}$, $W_{o}$ and bias $b_{i}$, $b_{o}$ represent the connection between two components respectively. The forget gate and the input gate together determine the current control cell status $C_{t}$:

(4) $$C_{t}=f_{t}*C_{t-1}+i_{t}*\tilde{C_{t}}$$

The output gate also generates a vector $o_{t}$ to determine the hidden state $h_{t}$ in the output state of the LSTM, as shown in the following equations:

(5) $$o_{t}=\sigma \left(W_{o}\cdot \left[h_{t-1},x_{t}\right]+b_{o}\right)$$

(6) $$h_{t}=o_{t}*\tan h\left(C_{t}\right)$$

In Equation (5), $W_{o}$ is the weighted matrix of the forget gate and $b_{o}$ represents the bias. In this study, the LSTM-AE network is adopted in this study to extract feature vectors of the heartbeat, the training parameters are feature number = 32, batch size = 128, epoch numbers = 100, and Adam optimizer is selected as the optimizer [25].

This research embeds the LSTM network into the AE framework; thus, the process of encoder and decoder is implemented by LSTM. The encoder converts the input $x_{t}$ to a hidden representation $h_{t}$ (feature vectors) using a deterministic mapping function:

(7) $$h_{t}=f\left(W\cdot \left[h_{t-1},x_{t}\right]+b\right)$$

where *W* is the weight between input $x_{t}$ and hidden representation $h_{t}$ and $h_{t}$ represents the bias. The decoder implements reconstructing the output $\hat{x_{t}}$ by $h_{t}$, which can be expressed as

(8) $$\hat{x_{t}}=f^{\prime }\left(W\prime \cdot h_{t}+b\prime \right)$$

where  W′ is the weight between hidden representation $h_{t}$ and output $\hat{x_{t}}$ and  b′ is the bias.

#### 3.2.2. K-Means Clustering Using Feature Vectors

The divided ECG heartbeats in each 30 min ECG segment are preliminarily clustered into K groups (K ≤ M, M represents the total number of heartbeats) based on the feature vectors using K-means clustering technique. In this study, K is determined by silhouette coefficient (SC):

(9) $$\mathrm{SC}=\frac{\sum_{i=1}^{M}\frac{b\left(i\right)-a\left(i\right)}{\max\left\{a\left(i\right),b\left(i\right)\right\}}}{M}$$

where a(i) and  b(i) are the intra-cluster dissimilarity and intercluster dissimilarity of *i*th coded feature, respectively. The maximum SC is defined as K.

#### 3.2.3. Template Construction and Template Classification

After K-means clustering, the distances between each coded feature sample in each group and its centroid are calculated, and sorted in ascending order Equation (10):

(10) sort_labelj=(∑i=1Nt|xi−aj|2)1≤j≤Kargsort

Where, $\mathrm{sort}\_{\mathrm{label}}_{j}$ is the index of the sample corresponding to the distance between the sample in group *j* and the centroid $a_{j}$ after sorted, and $N_{t}$ indicates the number of samples in the group.

The first 30 samples after sorting are selected to construct templates, and the type of each template is determined as PVC/Non_PVC based on the morphological rules referring to our previous work in [26]. In brief, three features (the QRS complex height, the QRS complex width, and the correlation coefficient of each template) and several prior-knowledge-based rules are used to determine the type of each template.

### 3.3. Heartbeat Classification

To quantify the similarity between each heartbeat waveform (*HW*) and the determined template waveform (*TW*), three characteristics are adopted in this study: cross-correlation coefficient (*Covr*), area difference (*ArDiff*) and energy difference (*EnDiff*). The *Covr* is defined as

(11) $$Covr\left(HW,TW\right)=\frac{\sum_{i=1}^{N}\left(HW_{i}-\bar{HW}\right)\left(TW_{i}-\bar{TW}\right)}{\sqrt{\sum_{i=1}^{N}{\left(HW_{i}-\bar{HW}\right)}^{2}\sum_{i=1}^{N}{\left(TW_{i}-\bar{TW}\right)}^{2}}}$$

where $\bar{HW}$ and $\bar{TW}$ are the mean values of *HW* and *TW*, respectively, *N* is the sample points of *HW* and *TW*. *ArDiff* indicates the area difference between *HW* and *TW*, the definition of *ArDiff* is

(12) $$ArDiff\left(HW,TW\right)=\frac{\left|\sum_{i=1}^{N}\left|HW_{i}\right|-\sum_{I=1}^{N}\left|TW_{i}\right|\right|}{\sum_{I=1}^{N}\left|TW_{i}\right|}\times 100\%$$

*EnDiff* is used to assess the energy difference between *HW* and *TW*, and is defined as

(13) $$\;EnDiff\left(HW,TW\right)=\frac{\sum_{i=1}^{N}{\left(HW_{i}-TW_{i}\right)}^{2}}{\sum_{i=1}^{N}{\left(TW_{i}\right)}^{2}}$$

The details of the proposed heartbeat classification are described as follows:

Step1: Evaluate the similarity between template and each intracluster heartbeats to determine the heartbeat type. If the current heartbeat and its related intracluster template meets the following conditions (14), the current heartbeat type and its template type are considered the same; else the current heartbeat is considered as “Unknown”.

(14) Covr≥0.9 or (Covr≥0.8 and ArDiff<10 and EnDiff<1)

Step2: Evaluate the similarity between “Unknown” heartbeat with all determined templates. The template matching result between “Unknown” heartbeat and all determined templates, as well as the rhythmic rules defined in [26] are considered simultaneously to identify the type of “Unknown” heartbeat.

For the long-term ECG signal in CPSC2020, the 24 h signal is divided into several 30 min segments, and the first 30 min segment is processed as described above. For other segments, a rule-based method is used to determine whether there is a need to update the template. If necessary, the previous described steps are performed to update the template; otherwise, the templates of the previous 30 min segment are used for the current 30 min segment.

### 3.4. Evaluation Method

Three common metrics including Se, P+ and ACC are used to evaluate the performance of the proposed method [27].

(15) $$\mathrm{ACC}=\frac{TP+TN}{TP+FP+TN+FN}\times 100\%$$

(16) $$\;\mathrm{Se}=\frac{TP}{TP+FN}\times 100\%$$

(17) $$\;P+=\frac{TP}{TP+FP}\times 100\%$$

where *TP* represents the number of PVC beats correctly identified; *TN* indicates the number of Non_PVC beats correctly identified; *FP* represents the number of Non_PVC beats incorrectly identified as PVC beats; *FN* indicates the number of PVC beats incorrectly identified as Non_PVC beats. Almost all experiments are carried out on Intel^®^Core™i5-8250U 1.60 GHz CPU and 8 GB RAM. The operating system is Windows10, the platform is Spyder3, and the deep learning tool Keras based on the Python programming language is used. However, the comparison of running time with the top five PVC scores of CPSC 2020 are carried out on Intel^®^ Xeon^®^ Silver 4215R 3.20 GHz CPU and 129 GB RAM with the help of the competition organizing committee. The operating system is CentOS Linux release 8.4.2105, the platform is Anaconda.

We adopt the scoring rules of the CPSC 2020 competition (PVC score) to evaluate the performance of the algorithm on the CPSC 2020 database, so that our algorithm can be compared with the participating teams of the cpsc2020 competition. The scoring rules are as follows.

- For a false positive (FP) detection, deduct 1 point.
- For a false negative (FN) detection, deduct 5 points, since from a clinical perspective, missed diagnosis is more serious than misdiagnosis, thus we penalize FN detection. The final score for PVC is the sum of all deducted points.

## 4. Results

### 4.1. Effectiveness of Feature Vectors Extracted by LSTM-AE

LSTM-AE model combines the LSTM network with the AE, which means the encoding and decoding process is performed by LSTM. Through LSTM, encoder extracts feature from the input ECG signal, while decoder implements the conversion from feature maps to the output. The parameters of the encoding and decoding operations are computed using unsupervised greedy training. In this paper, the input ECG signal of the LSTM-AE model is the raw ECG without filtering, while the loss function used to optimize the LSTM-AE model is calculated between the bandpass-filtered ECG signal and the reconstructed ECG signal. In order to determine the detailed hyperparameter (batch size and feature numbers) of the LSTM-AE model, we tested the PVC detection performance on different parameter settings. Table 2 illustrates the classification accuracy in MIT-BIH-AR database under different hyperparameter settings (take record 100 as an example), it can be seen that the model can provide better classification performance when batch size and feature numbers are set to 128 and 32, respectively. Therefore, the batch size and feature numbers are set to 128 and 32 in our paper, respectively.

**Figure 3:**
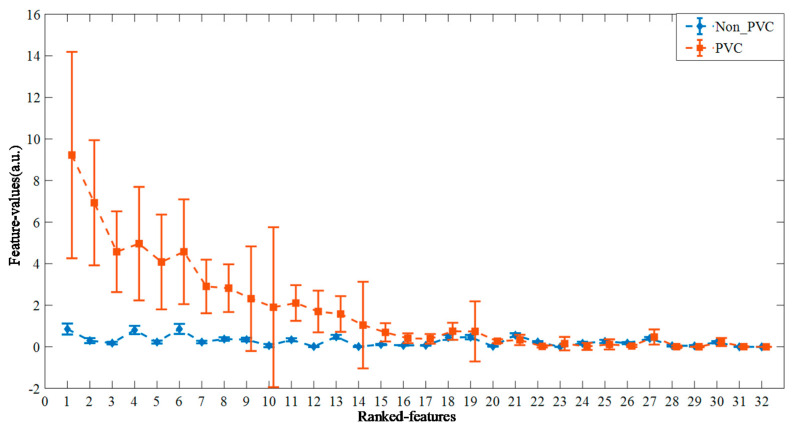
The ranked feature vectors of PVC and Non_PVC from record 228, according to the *t*-test *p*-value in ascending order.

Figure 3 shows the ranked feature vectors of PVC and Non_PVC in record 228 from the MIT-BIH-AR database, sorted according to their *t*-test *p*-value. It can be seen that the feature values of Non_PVC fluctuate slightly around 1, while the feature vectors of PVC vary greatly from 0 to 10. In addition, it is obvious that more than half the feature vectors between PVC and Non_PVC are different, which indicates that the feature vectors can substitute original ECG data for heartbeat clustering.

### 4.2. Results of K-Means Clustering

**Figure 4:**
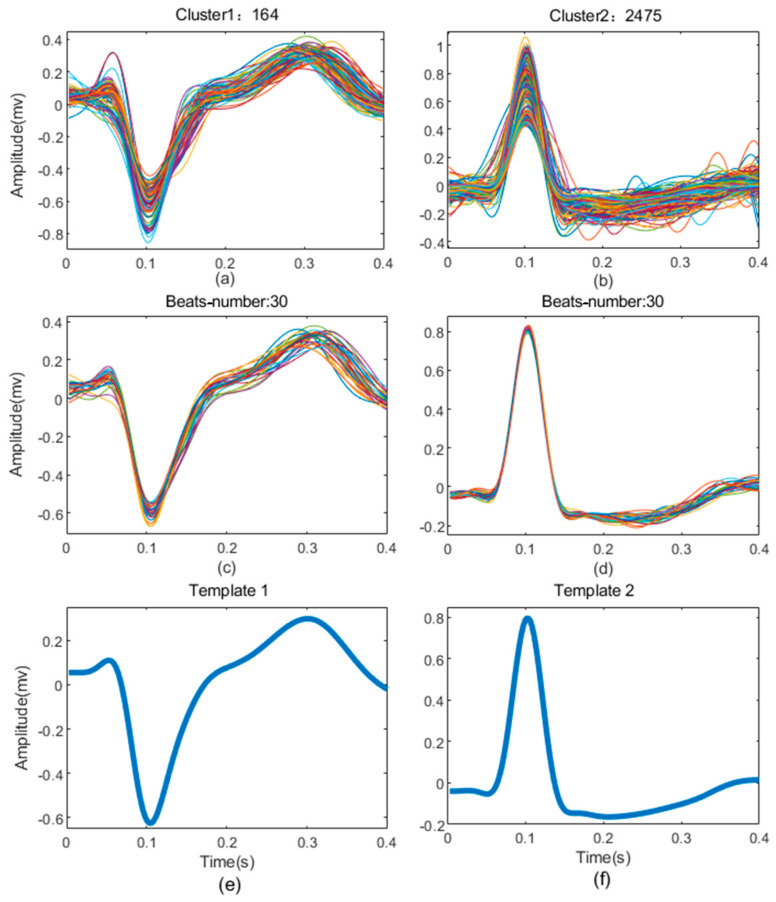
The results of clustering from record 210. (**a**,**b**) are all heartbeats superposition of each cluster; (**c**,**d**) are the 10 heartbeats extracted from each cluster to build templates; (**e**,**f**) are templates of the cluster.

The example of K-means clustering result of record 210 in MIT-BIH-AR database is shown in Figure 4. It can be seen that the heartbeats are clustered into only two groups (K = 2), including 164 heartbeats and 2475 heartbeats (Figure 4a,b), respectively. The heartbeats in each group show high similarity, and the templates (Figure 4e,f) constructed from the 30 heartbeats closest to the centroid of each group show great difference (Figure 4c,d). This demonstrates that the K-means clustering based on the feature vectors can better divide the heartbeats into different groups.

### 4.3. Results on MIT-BIH-AR Database

**Figure 5:**
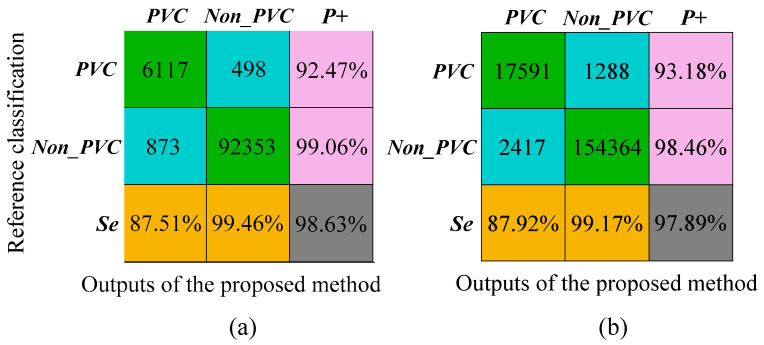
Results of the proposed method on the MIT-BIH-AR database and INCART database, respectively. (**a**) The evaluation indices of the proposed method on MIT-BIH-AR database; (**b**) the evaluation indices of the proposed method on INCART database.

Figure 5a shows the confusion matrix of the results on MIT-BIH-AR database, and the detailed results for this database are illustrated in the appendix (Table A1). The overall ACC is 98.63%, which is comparable to the state of art algorithms. The Se for Non_PVC and PVC beats is 99.46% and 87.51%, respectively; and the P+ is 99.06% and 92.47%, respectively. 

### 4.4. Results on INCART Database

The confusion matrix for the INCART database is shown in Figure 5b and the results for each recording are shown in the appendix (Table A2). For this database, we obtained a 97.89% overall ACC; Se 99.17% and P+ 98.46 % for non-PVC beats, and Se 87.92% and P+ 93.18% for PVC beats. In order to evaluate the multilead robustness of our method, the algorithm was independently verified in all 12-lead signals of the INCART database (Figure A1). The results on 12-lead INCART database indicated the proposed method had a good generalization ability between leads.

### 4.5. Results on CPSC2020 Dataset

Table 3 shows the results of the proposed method on CPSC 2020 dataset. According to the scoring standards of the competition, the PVC score reached 46,706 and 36,256 on the hidden dataset and training dataset, respectively. The result of our method is compared with the final scores of the top five teams on the hidden test set, we got first rank among the open-source codes. In addition, the computational complexity on the hidden test set is analyzed with the help of the competition organizing committee. Compared with the top five teams, the running time of our method is much shorter. It indicates that the proposed method has the potential to be applied in long-term dynamic ECG monitoring for PVC recognition.

## 5. Discussion

A PVC recognition algorithm based on integrating deep learning and rules was proposed in this study. Many ES-based or DL-based automatic ECG heartbeat classification algorithms have achieved high recognition results. However, they are complementary in terms of robustness and generalization.

The contribution of this paper is the combination of the DL-assisted template construction and ES-based heartbeat classification, which not only guarantees the accuracy but also improves the interpretability, robustness and generalization ability of the algorithm. A wavelet-based statistical process control (SPC) method was proposed for PVC recognition on MIT-BIH-AR database [28], the overall ACC was 97.90%, and the Se and P+ for PVC were 87.20% and 84.60%, respectively. This method could improve PVC sensitivity by manually adjusting parameter thresholds according to different situations, while our method could achieve high PVC sensitivity without any manual process. A real-time premature beat (PB) detection method for single-lead ECG was proposed based on several simple rules [26], which was reported to have low computational complexity and could be used for real-time PB detection for portable ambulatory ECG monitoring. However, their accuracy on the total data (85.56%) was still non-neglected for accurate clinical diagnosis. Malek et al. [29] developed an improved template matching technique for identifying normal and PVC beats in ECG signals, which was evaluated on the INCART, QT, MIT-BIH Supraventricular Arrhythmia, and Fantasia databases, and the accuracy was 97.91%, 99.34%, 99.89%, and 98.44%, respectively. One of the strengths of this method was the application of an adaptable threshold without the need for expert intervention, however, the features they adopted were more complex than ours. Talbi et al. [30] studied the effectiveness of the fractional linear prediction (FLP) technique on the ECG signal modeling, and developed a PVC recognition method based on the three coefficients of FLP and KNN, and the best accuracy of 96% was achieved on MIT-BIH-AR database. Most of the existing ES-based methods are efficient and requires less expert intervention, but the robustness still needs to be improved for daily life application.

From Table 4, we compared the PVC recognition between the proposed method with existing methods on MIT-BIH-AR database and INCART database. The satisfactory performance of the proposed method on these two clinical databases demonstrated that our method not only guarantees the accuracy and robustness advantages of DL-based method, but also improved the generalization capacity and interpretability advantages of ES-based methods.

With the popularity of machine learning, many researchers have implemented machine learning algorithms in arrhythmia recognition and achieved high performance. Mazidi et al. [32] designed a linear kernel-based SVM classifier with morphology, time domain, time-frequency domain and nonlinear features for PVC recognition, the method achieved a higher overall ACC and Se (99.78% and 99.91%, respectively) than our method. Wang et al. [34] proposed a PVC detection scheme based on image processing and CNN for scanned clinical ECG reports, and their Se and ACC could reach 95.47% and 98.25%, respectively. However, our method was unsupervised while the training set used in their method was overlapped in their test set. Oh et al. [12] proposed an automated system using a combination of CNN and LSTM for variable-length ECG classification (five class), they obtained the high classification accuracy of 98.10% without noise elimination on the MIT-BIH-AR database. The system could analyze ECG signals of different lengths with only a single type of arrhythmia, but it was computationally intensive. Yang et al. [27] applied stacked sparse autoencoders (SSAEs) and a Softmax regression (SF) for six types of ECG classification and achieved average 99.22% Se and 99.37% P+ on MIT-BIH-AR database. The features extracted by SSAE had no individual independent differences in feature selection and extraction accuracy, and almost no useful heartbeat information was lost. However, the method was semisupervised and required trained cardiologists to first classify each beat cluster into normal or ventricular. Therefore, it was inappropriate for analyzing long-term signals.

Although we did not participate in CPSC2020 as we were affiliated with the organizer of the challenge, the performance of the proposed method on long-term wearable ECG database (CPSC2020) was also compared with the published top five teams for PVC recognition in CPSC2020 (Table 3). The method proposed by the published champion team employed DenseNet model to classify the heartbeats into three categories (normal, premature ventricular contraction and supraventricular premature beat) and refined the results by a postprocessing procedure with several clinical rules. The algorithms of other teams were almost all DL-based methods, and they could achieve excellent performance on the training set, but they could not maintain such good results on the test set. The reason might be that these teams overoptimized the accuracy of their algorithm on the training set, leading to overfitting, which affected the algorithm results on hidden test set. Both our method and the published champion team’s results outperformed DL-based methods, indicating that the fusion of these two (ES-based and DL-based) methods had the potential to reform the existing methods based only on ES or DL.

To evaluate the computational complexity of our method, we computed and compared the operating time of our method and the CPSC2020 top five teams on the hidden test set. In addition, we also compared the running time with some published works in parallel. Three morphological features and seven statistical features were directly extracted, normalized and fed into CFNN classifier for PVC recognition, which could process 20-s segment within 2.1 s on a Samsung Galaxy J1 motherboard (a quad-core Cortex-A7 CPU clocked at up to 1.2 GHz with 1 GB RAM, OS Android 6.0) [15]. Khalaf et al. [37] proposed an SVM-based method on MATLAB R2010a on Intel^®^ Core™ i5 3.2 GHz processor and 8 GB RAM, and it consumed 54.8 ms for each beat classification. Arrais Junior et al. [38] reported an adaptive threshold and redundant discrete wavelet transform fusion method, which can process 30 min signals using only 61.2 s on the Matlab 2014a platform. These results showed that (1) the superposition of deep learning and time-frequency conversion processes will increase the complexity of the algorithm; (2) complex deep learning frameworks are indeed more time-consuming than simple CNN; (3) the DL-based feature extraction + ES-based postprocessing analysis generally take less time. The comparison results further verified the advantage of the fusion of these two (ES-based and DL-based) methods.

The employed DL-based method (LSTM-AE module) was used to extract features from ECG heartbeats for K-means clustering, and the PVC identification was based on a combination of multiple rules, including template matching and rhythm characteristics. The features used for classification are extracted according to the R-peak-relevant clinical experience: the Covr, ArDiff and EnDiff are used to map the morphological and frequency domain difference between PVC and Non_PVC, and the rhythmic rules are used to map the variation of RR intervals between PVC and Non_PVC. All these features are extracted only based on R peaks instead of those complex features detected from precise fiducial points (Q wave, S wave, etc.) and professional knowledge, which can not only retain the interpretability of the proposed algorithm, but also improve the antinoise ability of the algorithm.

Although the proposed method is an important contribution to unsupervised PVC identification, there are three main limitations. (1) The performance is affected by the misalignment of QRS complex, more accurate QRS detection algorithm should be designed to detect the peak of each QRS complex for precise ECG classification. (2) This method is trained and tested only on the Windows platform, so further work is needed to embed the algorithm to the mobile terminal for daily life monitoring application. (3) Only one-channel information is considered in this paper, multichannel information should be considered from multilead ECG monitoring systems for accuracy improvement of PVC recognition, or even other kinds of heartbeat classification.

## 6. Conclusions

In summary, an unsupervised adaptive PVC recognition algorithm is proposed for single-lead ECG based on a novel expert system and deep learning combination strategy. The personalized heartbeat templates are firstly clustered by K-means using LSTM-AE extracted features and determined by rule-based methods. Then, each heartbeat is classified into PVC or Non_PVC by a series of rules. The performance of the proposed algorithm is tested on the clinical databases (MIT-BIH database and INCART database) and long-term wearable databases (CPSC2020 training set and hidden test set). The comparison with the existing PVC algorithms shows that the proposed method embraces the advantages of deep learning and rules, and achieves high accuracy, robustness, and interpretability.

## Figures and Tables

**Table 1:** The Detailed Information of Three Database.

	Database	ECG Length	# PVC Beats	# Non_PVC Beats	# Total Beats	Sampling Frequency (Hz)
Training	MIT-BIH ^1^	30 min	6990	92,851	99,841	360
Test	INCART-12	30 min	20,008	155,652	175,660	275
	CPSC2020 Training	~24 h	42,075	853,636	895,711	400

^1^ Four records (102, 104, 107, and 217) containing paced beats in MIT-BIH database were excluded in this study. # means the number of each beats.

**Table 2:** The example of classification accuracy in MIT-BIH-AR database under different hyperparameter setting (record 100).

	Batch	64	128	256
Feature Numbers				
16		99.62%	99.65%	98.61%
32		99.68%	**99.78%**	98.59%
64		99.33%	99.60%	99.65%

**Table 3:** Detailed information on three databases.

Code No.	CPSC1077 ^1^	CPSC1091	CPSC1093	CPSC1082	CPSC1089	This Work
Method	DenseNet + Rules	DL-based ^2^ +Rules	Bidirectional LSTM	WT + DL-based ^3^	CNN	LSTM-AE + K-Means + Rules
PVC Score of Test	41,479	55,706	95,900	97,913	142,228	46,706
PVC Score of Training	-	16,467	6370	4482	11,086	36,256
Running Time (s)	1600.35 ± 311.32	695.55 ± 185.45	12,810.90 ± 726.48	18,260.57 ± 2100.84	368.29 ± 33.27	215.93 ± 59.32

^1^ This team did not publish their code, so we could not obtain the evaluation score of their algorithm on the training set. The other codes are available in http://2020.icbeb.org/CSPC2020 (accessed on 17 March 2022). ^2^ This DL-based method refers to a deep learning architecture containing multi-dilated convolutional blocks and a squeeze-and-excitation network. ^3^ This DL-based method refers to the combination of one-dimensional convolutional layers and gated recurrent unit layers.

**Table 4:** Comparison of PVC recognition between the proposed method and existing methods on MIT-BIH-AR database and INCART database.

Author	Class and Focus	Method	Database	# Total Beats	# PVC Beats	Se (%)	P+ (%)	ACC (%)
Talbi et al., 2016 [30]	PVC, Non_PVC	KNN + FLP	MIT-BIH-AR	95,743	7147	80.88	-	94.63
Wang et al., 2017 [31]	PVC, Non_PVC	Statistics +SVM		110,906	-	75.00	-	93.13
Jung et al., 2017 [28]	PVC, Non_PVC	Wavelet-based SPC		-	-	87.20	84.60	97.90
Mazidi et al., 2019 [32]	PVC, Non_PVC	SVM		82,163	7111	99.91	-	99.78
Li et al., 2019 [33]	PVC, Non_PVC	Wavelet Transform		100,372	6990	82.55	82.39	97.56
Cai et al., 2020 [26]	Normal, PAC, PVC	+CNN		98,426	6734	76.54	90.47	85.56
Kalidas et al., 2020 [19]	PVC, Non_PVC	Rules		93,432	6898	96.58	97.20	-
Wang et al., 2021 [34]	PVC, Non_PVC	SSAE + Random Forests		24,922	2187	95.47	98.75	98.25
This study. 2021	PVC, Non_PVC	OTSU + CNN		99,841	6990	87.51	92.47	98.63
Li et al., 2013 [8]	PVC, Non_PVC	LSTM-AE + K-Means+	INCART	175,892	20,011	93.40	66.50	94.00
Oster et al., 2015 [35]	PVC, Non_PVC	Rules		175,871	20,011	95.40	99.30	-
Rahhal et al., 2018 [36]	Normal, PVC and Others	Template-matching		-	-	85.20	80.90	92.00
Kalidas et al., 2020 [19]	PVC, Non_PVC	SKF with X-factor Mode		175,674	19,990	88.08	94.70	-
This study. 2021	PVC, Non_PVC	SDAEs + DNN		175,660	20,008	87.92	93.18	97.89

# means the number of each beat.

**Figure A1:**
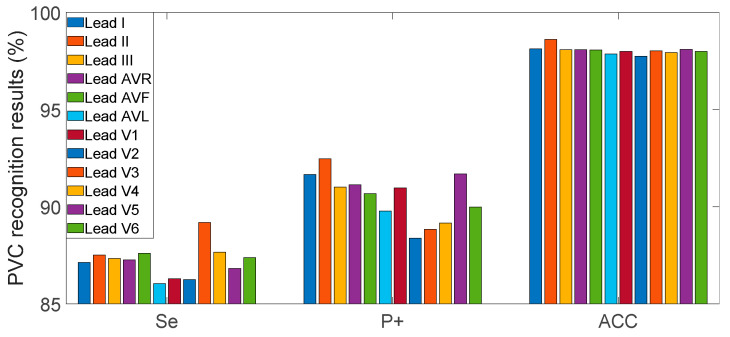
Evaluation indices of the proposed method in 12-lead INCART database.

## Data Availability

The MIT-BIH-AR database presented in this study is openly available in [physionet] at [10.1109/51.932724 and 10.1161/01.CIR.101.23.e215], the corresponding webpage is “https://www.physionet.org/content/mitdb/1.0.0/” (accessed on 27 February 2022); the INCART database is openly available in [physionet] at [10.1161/01.CIR.101.23.e215], the corresponding webpage is “https://www.physionet.org/content/incartdb/1.0.0/” (accessed on 27 February 2022); the training data of CPSC2020 database is openly available at [10.1166/jmihi.2020.3289], the corresponding webpage is “http://2020.icbeb.org/CSPC2020/” (accessed on 27 February 2022).

## Appendix A

**Table A1:** PVC recognition results on the MIT-BIH-AR database.

Record	Se (%)	P+ (%)	ACC (%)	Record	Se (%)	P+ (%)	ACC (%)
100	100.00	100.00	100.00	202	94.74	81.82	99.77
101	-	-	100.00 ^1^	203	73.76	91.06	95.00
103	-	-	100.00 ^1^	205	92.96	100.00	99.81
105	90.24	68.52	99.18	207	65.07	61.54	91.50
106	79.81	100.00	94.82	208	92.42	100.00	97.08
108	88.24	65.22	99.43	209	100.00	100.00	100.00
109	76.32	100.00	99.64	210	75.77	96.71	98.03
111	100.00	4.35	98.96	212	-	-	100.00 ^1^
112	-	-	100.00 ^1^	213	98.18	99.08	99.79
113	-	-	100.00 ^1^	214	60.78	100.00	95.57
114	95.35	100.00	99.89	215	91.46	100.00	99.58
115	-	-	100.00 ^1^	219	79.69	100.00	99.40
116	91.67	100.00	99.62	220	-	-	100.00 ^1^
117	-	-	100.00a	221	97.22	100.00	99.55
118	93.75	40.54	98.99	222	-	0.00	88.99 ^2^
119	99.55	100.00	99.90	223	63.21	100.00	93.28
121	100.00	100.00	100.00	228	98.62	100.00	99.76
122	-	-	100.00a	230	100.00	100.00	100.00
123	100.00	100.00	100.00	231	100.00	100.00	100.00
124	78.72	100.00	99.38	232	0.00	-	99.89 ^2^
200	94.97	99.74	98.34	233	94.10	99.74	98.34
201	99.49	89.95	98.83	234	100.00	100.00	100.00

^1^ This single record excludes PVC beats, and there is no false detection of PVC beats. Therefore, the TP, FN, and FP of this record are all 0. ^2^ This single record excludes PVC beats but false detects Non_PVC beats as PVC beats. Therefore, TP and FN of this record are 0, but TP is not 0.

**Table A2:** PVC recognition results on the INCART database.

ID	Se (%)	P+ (%)	ACC (%)	ID	Se (%)	P+ (%)	ACC (%)	ID	Se (%)	P+ (%)	ACC (%)
I01	100.00	86.00	97.97	I26	25.00	50.00	99.73	I51	97.63	100.00	99.32
I02	87.34	94.34	98.47	I27	100.00	100.00	100.00	I52	100.00	100.00	100.00
I03	92.00	100.00	99.59	I28	75.00	33.33	99.59	I53	96.94	100.00	98.50
I04	22.31	93.10	96.01	I29	68.33	99.63	90.45	I54	68.18	93.75	99.66
I05	83.40	99.52	97.62	I30	80.13	99.83	93.86	I55	94.12	100.00	99.95
I06	100.00	81.82	99.92	I31	70.99	99.28	87.44	I56	100.00	100.00	100.00
I07	100.00	5.88	99.41	I32	84.21	97.96	99.38	I57	100.00	48.84	99.23
I08	86.61	99.02	97.65	I33	100.00	16.67	99.73	I58	100.00	100.00	100.00
I09	73.17	83.33	99.43	I34	-	0.00	99.03	I59	64.20	96.30	98.56
I10	83.13	100.00	99.62	I35	77.46	100.00	97.18	I60	-	0.00	98.87 ^2^
I11	100.00	50.00	99.81	I36	86.89	100.00	98.49	I61	-	-	100.00 ^1^
I12	33.33	14.29	99.43	I37	99.56	100.00	99.92	I62	32.45	100.00	76.21
I13	100.00	100.00	100.00	I38	86.61	100.00	97.29	I63	58.70	100.00	97.13
I14	100.00	100.00	100.00	I39	94.25	100.00	98.99	I64	69.57	100.00	99.63
I15	33.33	50.00	99.89	I40	92.39	92.39	99.47	I65	93.46	100.00	99.06
I16	100.00	50.00	99.87	I41	100.00	33.33	99.88	I66	97.50	100.00	99.79
I17	92.59	100.00	99.88	I42	99.29	99.87	99.58	I67	97.93	100.00	99.63
I18	91.80	99.70	98.98	I43	97.86	99.91	98.87	I68	95.65	99.35	99.70
I19	84.59	100.00	93.65	I44	100.00	100.00	100.00	I69	99.40	98.81	99.86
I20	75.45	100.00	98.98	I45	100.00	100.00	100.00	I70	-	0.00	92.50 ^2^
I21	87.50	77.78	99.86	I46	98.34	99.76	99.70	I71	-	0.00	86.22 ^2^
I22	69.73	99.23	98.18	I47	98.92	96.84	99.80	I72	91.19	33.85	68.17
I23	61.54	100.00	99.77	I48	98.72	100.00	99.87	I73	94.29	100.00	99.80
I24	16.67	50.00	99.77	I49	100.00	96.43	99.95	I74	98.18	100.00	99.79
I25	60.00	37.50	99.59	I50	50.00	50.00	99.87	I75	99.02	100.00	99.71

^1^ This single record excludes PVC beats, and there is no false detection of PVC beats. Therefore, the TP, FN, and FP of this record are all 0. ^2^ This single record excludes PVC beats but false detects Non_PVC beats as PVC beats. Therefore, TP and FN of this record are 0, but TP is not 0.
